# Effects of Differences in Fibre Composition and Maturity of Forage-Based Diets on the Microbial Ecosystem and Its Activity in Equine Caecum and Colon Digesta and Faeces

**DOI:** 10.3390/ani11082337

**Published:** 2021-08-08

**Authors:** Sara Muhonen, Sophie Sadet-Bourgeteau, Véronique Julliand

**Affiliations:** 1 AgroSup Dijon, Univ. Bourgogne Franche-Comté, PAM UMR A 02.102, 21000 Dijon, France; veronique.julliand@agrosupdijon.fr; 2 AgroSup Dijon, INRAE, UMR AgroEcologie, BP 87999, CEDEX, 21079 Dijon, France; sophie.bourgeteau-sadet@agrosupdijon.fr

**Keywords:** forage fibre, hindgut microbiota, grass, lucerne, digestion

## Abstract

**Simple Summary:**

Horses are herbivores and forage-based diets are a natural choice for them. Traditionally, horse diets have included a large portion of cereals and have been associated with different intestinal problems. Feeding more forage and less concentrate has been shown to promote both physical and mental health and performance in horses. However, the nutritional quality of forage can differ greatly. This study compared the effect of two different forage diets and the more conventional forage and concentrate diets, on the hindgut microorganisms and the environment. No differences were found between the three diets in the concentration of total bacteria, fungi and protozoa, of cellulose-utilising bacteria or in the concentration of short-chain fatty acids. It can be concluded that a forage diet which fulfils the energy and protein requirements without having to add starch rich concentrate can benefit hindgut health. In addition, further studies on plant-fibre and forage diets for horses are of great importance for horse feeding, for advisors, veterinarians and for the diet formulations industry.

**Abstract:**

Fibrous feeds are essential for horses. When developing feeding regimens promoting health and performance, we need to understand the digestion of plant cell walls and the functioning of the hindgut microbial ecosystem. Our objective was to investigate the effect of grass fibre maturity and legume forage on the hindgut microbiota and its activity. Six caecum and colon fistulated geldings were fed three diets differing in fibre composition: concentrate and late harvested grass haylage (35:65 energy ratio) (C); early and late harvested grass haylage (80:20) (G); lucerne and late harvested grass haylage (80:20) (L) for 28 days in a Latin-square design. No differences were measured in total bacteria concentrations, fungi and protozoa numbers nor in cellulolytic bacteria concentrations between the diets. Short-chain fatty acid concentrations did not differ between diets, but a lower (acetate + butyrate)/propionate ratio when the horses were fed Diet C, compared to G and L, was observed, suggesting lower fibrolytic and higher amylolytic activity. The pH increased when the horses were fed Diet L and decreased when fed C and G from caecum to faeces. The buffering capacity (BC) of hindgut digesta was five to fifteen-fold higher than that of the feeds, suggesting a decreased effect of feed BC as digesta travelled through the digestive tract. In conclusion, an early harvested forage opens up the possibility for forage-only diets, providing high energy without the negative effects of concentrate.

## 1. Introduction

Horses are large herbivorous hindgut fermenters and need to eat fibrous feeds. However, traditionally the diets of equine athletes have included a large portion of cereals and have been associated with various intestinal problems. Thus, recent advances in equine nutrition research recommend more plant-fibre and forage-based diets for horses [1]. To develop modern feeding regimens, it is essential to examine in depth the complex dynamics between the digestion of their natural fibrous feeds and the hindgut ecosystem of these herbivores.

Mature forages are less digestible and nutritive value decreases as the cell wall content increases with advancing stages of maturity [2,3]. Hence, forage maturity affects the extent of forage fibre digestion. Generally, legumes contain less fibre but also more lignified and less digestible fibre than grasses [4]. Legume forages, such as lucerne, are high in pectin, which is the most rapidly degraded complex carbohydrate and is not fermented to lactate [5]. Extensive differences in ruminal degradation kinetics have been shown between legumes and grasses [6].

*Ruminococcus flavefaciens* and *Fibrobacter succinogenes* have been identified as the main cellulolytic bacterial species in the equine [7,8]. Fungi also appear to be strong cellulose degraders [9,10], but no closer examination has been done in vivo comparing fungal effects with diets different in fibre quality. Protozoa have been suggested not to play an important role in cellulose digestion in horses [11]. However, recent studies showed that caecum ciliate protozoa produce enzymes capable of degrading cellulose [12,13] and large fibrolytic protozoa may to some degree support forage digestion [13,14]. Both bacteria and protozoa in equine caecal content have been shown to be involved in the degradation of hemicellulose and pectin [13,15]. Microbial composition and activity between large intestinal compartments and faeces have been shown to differ [10,12]. The use of faeces for representing hindgut content in terms of the microbial ecosystem is important but may be questionable. The inter-segment variation has been suggested to be stronger than the inter-animal variation [16].

Two studies comparing diets with different levels of fibre and starch have shown no effect on cellulolytic bacteria concentration in the pony caecum [11,17] and colon [11], whereas another one reported higher cellulolytic bacteria counts throughout the digestive tract of horses on a high fibre diet [18]. However, the quality of the fibre probably has a greater impact on the enzymatic activity of cellulose bacteria and thus SCFA production [17,18]. Dietary differences in fibre and starch affect SCFA and lactate concentrations and the hindgut digesta’s capacity to buffer lactate has a positive impact on the digesta pH and hindgut health of the horse. Different regimens and dietary fibre compositions have been suggested to have an effect on the buffering capacity (BC) in the equine gastrointestinal tract [19,20]. The BC of feeds depends on the cation exchange capacity of the fibre and, to a lesser extent, on the fermentation of protein to ammonia [5]. Mature legume forages are the most effective whereas mature grasses are poor at supplying exchangeable BC [5]. To our knowledge, the BC of caecum and colon content in young and mature grass and legume diets has not been examined.

How differences in grass fibre maturity and legume forage affect the ecosystem of the equine hindgut is not well documented. The objective of this study was to examine the effect of feeding young grass haylage or lucerne haylage compared to the more conventional mature grass haylage and concentrate diet on the microbial ecosystem, its activity and the BC of the equine hindgut and faeces.

## 2. Materials and Methods

The experiment was performed at AgroSup Dijon, France, and was approved by the Burgundy University ethical committee (agreement no. B0810).

### 2.1. Animals and Design

Six adult crossbreed geldings, aged 7 to 15 years, with body weights (BW) between 440 to 493 kg, were used. They were fistulated in the caecum and the right ventral (RV) colon. Horses were kept in individual free stalls on artificial bedding (TIERWOHLTM), had ad libitum access to water, had access to a paddock (10 × 20 m) with no grass for 1 h 5 days per week and were exercised in a horse walker (approximately 7 km/h) for 1 h three times per week. Horses were dewormed (Eraquell Ivermectine, Ceva, Libourne, France) 22 days before the start of the experiment. The six horses were randomly assigned to three diets in a Latin square design. The three experimental periods were 28 days long and the first 5 days comprised a stepwise change in diet.

### 2.2. Diets

The forages used in this experiment were two grass haylages that were the first cuts produced in the same field but at different stages of maturity (harvested 6 weeks apart) and a lucerne haylage. The grass haylages were dominated by timothy and ryegrass with a seed mixture of: 21% timothy, 17% meadow fescue, 23% Braun’s festulolium, 17% perennial ryegrass, 10% orchard grass, 7% hybrid ryegrass, 5% white clover. Half of the grass ley was harvested as the early grass haylage cut on 31 May and the other half of the ley was harvested as the late grass haylage cut on 13 July outside Stockholm, Sweden (Lat. 59° N., Long. 17° E.). The two first cuts at two different stages of maturity resulted in in vitro digestible organic matter (IVDOM) values of 89% and 63% for the early and the late harvest, respectively. The difference in maturity was also apparent in metabolisable energy (ME) and chemical composition: 11.6 MJ, 172 g crude protein (CP), 280 g crude fibre, 521 g neutral detergent fibre (NDF), 299 g acid detergent fibre (ADF), 22 g acid detergent lignin (ADL)/kg DM, and 7.5 MJ, 80 g CP, 374 g crude fibre, 670 g NDF, 409 g ADF, 50 g ADL/kg DM for the early and the late harvest, respectively. The lucerne (*Medicago sativa*) haylage was a second cut, harvested in early bloom on 11 August 2009 in Alsace, France (Lat. 47° N., Long. 7° E.) (inclusion of weeds was estimated to be approximately 10%). The lucerne IVDOM was 65% and the ME and chemical composition was: 8.7 MJ, 157 g CP, 350 g crude fibre, 483 g NDF, 382 g ADF and 77 g ADL/kg DM.

Three diets were fed restrictedly and the feeds were mixed to result in similar DM, ME and CP intakes: the conventional diet with concentrate (rolled oats, soybean meal) and late harvested grass haylage with a 35:65 energy ratio, id est, 35% of the energy intake was provided by concentrate and 65% was provided by late harvested grass (Diet C), the grass diet with early harvested and late harvested grass haylage with an 80:20 energy ratio (+small amount soybean meal) (Diet G) and the legume diet with lucerne haylage and late harvested grass haylage with an 80:20 energy ratio (Diet L) (Table 1). The conventional diet with concentrate provided 1.9 to 2.0 kg concentrate per day. For the grass and legume diet the forage of interest constituted the main body of the diet and provided 80% of the energy intake. The individual diets were also calculated to meet the maintenance energy (0.5 MJ ME × BW0.75), mineral and electrolyte requirements [21]. All horses were offered more than 1 kg DM of forage/100 kg BW daily. Diets were supplemented (65 g/day) with a commercial mineral product (Krafft, Falkenberg, Sweden) and salt (17 to 19 g/day). Additional chalk (Calcium carbonate) was added to Diets C and G to ensure isocalcium intake.

The horses were fed approximately 20% of the daily feed allowance at 08.00 h, 20% at 10.00 h, 20% at 16.00 h and 40% at 17.30 h. The feeding occasions were spread throughout the day to avoid prolonged periods without foraging opportunity [1] but were also adjusted for the working schedule at the animal facility. The same ratios, concentrate:forage or forage:forage, were fed at each meal.

### 2.3. Feed Sampling and Analyses

Feed samples for chemical analyses were taken during 3 days at the end of each period and haylage samples were immediately frozen (−20 °C). The samples were pooled into one sample for each feed and period (total volume of at least 9 L for forage samples and of at least 1.5 L for concentrate samples), dried in an air-forced oven at 70 °C until constant weight and ground with a hammer mill (1 mm screen).

DM, ash, CP, crude fat and gross energy were determined by the ISO standard procedure 6496 (1999), 5984 (2002), 5983 (2005), 6492 (1999) and 9831 (1998), respectively. Crude fibre, NDF, ADF and ADL were analysed according to standard procedures ISO 6865 (2000), ISO/DIS 16472 (2005) and ISO 13906 (2008), respectively. Starch and water soluble carbohydrates (WSC) were analysed according to standard procedures [23]. Dried and milled samples of haylage were incubated for 96 h in buffered rumen liquid (39 °C), and IVDOM was determined [22]. Minerals were determined by boiling samples in nitric acid (7 M) and measurements were performed with Inductively Coupled Plasma Optical Emission Spectrometry (SS-EN 14538:2006, Ametek Spectro, Kleve, Germany). BC was measured on dried and milled haylage samples that were suspended in distilled water and titrated down to pH 4 with lactic acid [24].

### 2.4. Caecum, Right Ventral Colon and Faecal Sampling and Measures

At the end of each experimental period, samples for cultivable microbial analysis were obtained by gravity, via the caecum and RV colon cannulas and grab-samples were taken from rectum into CO_2_-saturated flasks maintained at 38 °C. Sampling was performed at 12.00 h on day 28. The experiment started with two horses per day for 3 days to enable the sampling and handling of the cultivable microbial analysis directly upon arrival at the laboratory. Samples for chemical and molecular microbial analyses and BC were taken from the caecum, RV colon and rectum at the same time as the samples for cultivable microbial analysis. Caecum and RV colon content and faeces for chemical analyses were filtered (squeezed) through a 100-μm Blutex nylon screen (SAATI, Sailly-Saillisel, France) and two sub-samples were frozen (−20 °C) pending analyses. Caecum and RV colon content and faeces for molecular microbial analyses were frozen (−80 °C) pending analyses. The caecal and RV colonic content pH and faecal pH were measured immediately after each collection, using an electronic pH meter (WTW, Weilheim, Germany). The caecal, colonic and faecal fluids were analysed for SCFA according to standard procedures [25], and for D- and L-lactate using K-DLATE 12/12, Megazyme, Wicklow, Ireland. BC was measured on dried and milled samples of caecum and colon digesta and faeces that were suspended in distilled water and titrated down to pH 4 with lactic acid [24].

### 2.5. Microbial Analyses

Bacterial inoculations were conducted in three dilutions with four replicates and were performed under an O_2_-free CO_2_ gas phase. Total viable anaerobic bacteria were cultivated for 48 h at 38 °C in roll tubes prepared with a modified complete agar medium [7,26]. Cellulolytic bacteria were cultivated with a broth medium [7,27] for 14 days at 38 °C and concentrations were calculated by the most probable number method [28]. Xylanolytic and pectinolytic bacteria counts were determined in anaerobic roll tubes for 48 h at 38 °C on a selective medium [26] adapted and modified in our laboratory at AgroSup Dijon [29]. Lactic acid-utilising bacteria were selectively cultivated in roll tubes for 48 h at 38 °C [30]. Amylolytic bacteria were enumerated with an overlay method on Petri plates incubated for 48 h at 38 °C [31].

Total DNA was extracted as previously described [32]; 0.5 mL of caecum and colon content and 0.25 g of faeces were used. Extracted DNA samples were stored in Eppendorf tubes at −20 °C until processing. Microbial DNA from total bacteria, *Fibrobacter succinogenes*, *Ruminococcus flavefaciens*, *Ruminococcus albus*, fungi and protozoa were amplified by PCR with different primer sets (Table 2). The reaction mixture (final volume 25 µL) for the Real-time qPCR contained forward and reverse primers (10 µM of each), 1 × iQ SYBR Green Supermix (BioRad, Marne-La-Coquette, France) and 1.5 ng DNA template. The BioRad iCycler iQ5 (BioRad, Marne-La-Coquette, France) with fluorescence detection of SYBR green dye was used for the DNA amplification. Amplification conditions for total bacteria, *F. succinogenes*, *R. flavefaciens*, *R. albus*, fungi and protozoa were: 1 cycle at 95 °C for 3 min for initial denaturation, followed by 45 cycles of 95 °C for 30 s, 60 °C for 30 s and 72 °C for 1 min. Amplification was followed by the melting curve program (72–95 °C for 23 min with a continuous fluorescence measurement). Each DNA sample was run in duplicate and for every assay a negative control without a DNA template was included. A threshold cycle value (Ct) was determined for each measurement, defined as the number of cycles necessary to reach a point at which the fluorescence signal was first recorded as statistically significant above background. The Ct was determined with a baseline set manually at 100 relative fluorescence units. For total bacteria, *F. succinogenes*, *R. flavefaciens* and *R.albus* qPCR counting, an absolute quantification was conducted using standard curves. Standards were produced with pure cultures (*Butyrivibrio fibrisolvens*, *F. succinogenes* S85, *R. flavefaciens* C94 and *R. albus* 7, respectively), colony counts were conducted and DNA was extracted from 1 mL of adapted broth medium. Standard curves were generated from serial dilutions of a known concentration of genomic DNA from each species and the Ct was plotted against bacterial quantity (colony forming units (CFU)). The total number of bacteria (CFU) was interpolated from the averaged standard curves as previously described [33]. Fungi and protozoa were presented as the N-fold microbe expression difference between diets: Fold changes = 2 exp(∆∆Ct) with ∆∆Ct = mean(∆Ct)2 − mean(∆Ct)1, where (∆Ct)i is the mean of the microbe normalized Ct of the different samples of condition i.

### 2.6. Statistical Analysis

When performing experiments with animals, the number of individuals shall be restricted to the smallest number in accordance with the principle of the three Rs (Replacement, Reduction and Refinement). Using fistulated animals, especially when it comes to equines fistulated in both the caecum and RV colon, it is not possible to obtain a large number of animals. However, previous trials with four caecum and RV colon fistulated horses in change-over designs proved sufficient for finding statistically significant differences examining the hindgut microbiota and its activity [37,38,39,40]. Thus, we estimated that six caecum and RV colon fistulated horses in a Latin square design was a reasonable sample size to detect significant changes. During experiment, one horse had to be excluded and the statistical analysis was therefore performed on five horses.

For cultured bacteria concentrations, logarithmic transformations (log10) were performed on colony counts before the statistical analysis. Analysis of variance was performed using the PROC MIXED of the SAS software (SAS Inst., Inc., Cary, NC, USA). All variables were analysed by a statistical model including fixed (period, diet, segment (caecum, RV colon and faeces)) and random (horse) effects. The model components were the overall mean, the effect of horse, the effect of period, the effect of segment, the effect of diet, the effect of the interaction between diet and segment, and the random error. For fungi and protozoa ANOVA was performed with one diet as a point of comparison and to compare the three segments. Pair-wise t-tests were used to separate the main effect means. Values are presented as least square means with the pooled standard error of the mean (SEM). Differences were considered statistically significant at *p* < 0.05.

## 3. Results

### 3.1. Feed Intake

One horse was excluded from the experiment due to a colic on the concentrate diet and the statistical analysis was therefore performed on five horses. There were no feed refusals and feed intakes of g/100 kg BW are shown in Table 1. The three diets differed in DM and energy intake with a lower DM intake (200 g/100 kg BW and day) in Diet G and a higher energy intake (0.8 MJ/100 kg BW and day) in Diet G than in Diet L. Diet G provided the lowest and Diet L the highest intake of crude fibre, ADF, ADL and cellulose. The NDF and NSC intake was lower in Diet G and the hemicellulose intake was highest in Diet C and lowest in Diet L. The NSP intake was higher in Diet L, which was comprised of the legume, the lucerne haylage, and probably also implied the highest intake of pectins.

### 3.2. Cultured Bacterial Flora

Total viable anaerobic bacteria did not differ between diets (Table 3) but counts were higher in the caecum than in the RV colon with the faeces being intermediate (caecum: 8.2 log CFU/mL, colon: 7.3 log CFU/mL, faeces: 7.8 log CFU/g, SEM = 0.22, *p* = 0.042). Cellulolytic bacteria did not differ between diets (Table 3). Lactate-utilising bacteria were higher when the horses were fed Diet C compared to Diet G and L (Table 3) and were lower in the colon than in the caecum and faeces (caecum: 7.0 log CFU/mL, colon: 6.3 log CFU/mL, faeces: 7.1 log CFU/g, SEM = 0.17, *p* = 0.020). Xylanolytic and pectinolytic bacteria had higher counts when the horses were fed Diet C compared to Diet G and L (Table 3). Xylanolytic bacteria were lower in the colon than in the caecum and faeces (caecum: 7.4 log CFU/mL, colon: 6.6 log CFU/mL, faeces: 7.4 log CFU/g, SEM = 0.17, *p* = 0.027). There was a tendency (*p* = 0.057) for pectinolytic bacteria to show the same pattern with lower counts in the colon (colon 6.9 log CFU/mL vs. 7.5 log CFU/mL and g in caecum and faeces, respectively, SEM = 0.16).

### 3.3. Bacterial Flora Using Real-Time qPCR Analysis

**Figure 1:**
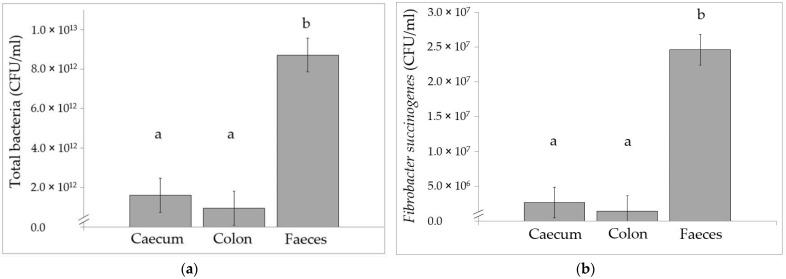

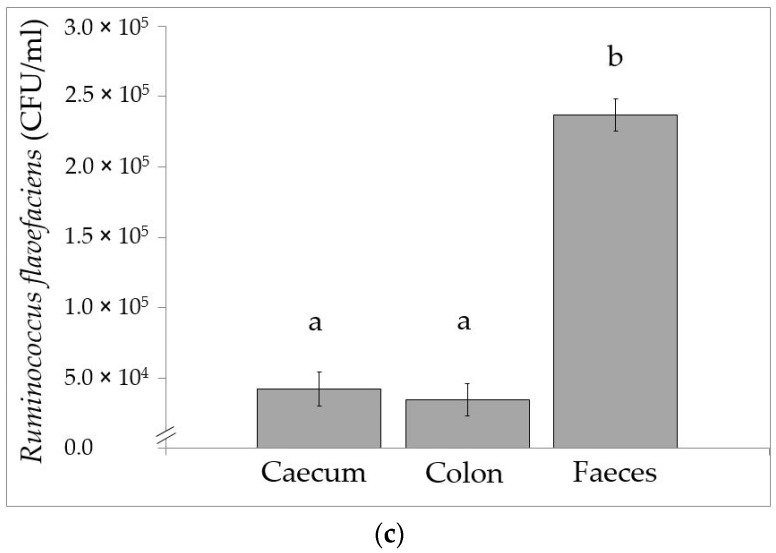
Equine caecal, colon (right ventral) and faecal concentrations of (**a**) total bacteria (**b**) *Fibrobacter succinogenes* and (**c**) *Ruminococcus flavefaciens* after three weeks of adaptation to forage based diets differing in fibre composition and maturity. There was an effect of segment (*p* < 0.001). Since there were no effect of diet and no diet × segment interactions, least square means across diets (with the pooled standard error of the mean) are presented (*n* 5). ^a,b^ Mean values with unlike superscript letters were significantly different (*p* < 0.05). Diet C: concentrate (oats, soybean meal) and late harvested grass haylage (35:65 energy ratio), Diet G: early and late harvested grass haylage (80:20) (+small amount soybean meal), Diet L: lucerne haylage and late harvested grass haylage (80:20).

Absolute values of total bacteria, *F. succinogenes* and *R. flavefaciens* did not differ between diets, but concentrations were higher in the faeces than in the caecum and colon content (Figure 1). *R. albus* was not detected in the caecum and colon content but was detected in faeces. Concentrations of faecal *R. albus* did not differ (*p* = 0.288) between diets, and averaged 1.1 × 10^5^ CFU/mL, 4.1 × 10^5^ CFU/mL and 6.3 × 10^4^ CFU/mL for Diet C, G and L, respectively, SEM = 1.3 × 10^5^.

### 3.4. Fungi and Protozoa Using Real-Time qPCR Analysis

Relative quantifications of fungi did not differ between diets but concentrations were higher in faeces than in the caecum and the RV colon when Diets C and G were compared (Table 4). Relative quantifications of protozoa did not differ between diets or segments (Table 4).

### 3.5. Short-Chain Fatty Acids, pH and Buffering Capacity

The concentrations of total SCFA, acetate, propionate and butyrate in caecum, colon and faeces did not differ between diets and were lower in faeces than in the caecum and colon (Table 5). The (acetate + butyrate)/propionate ratio did not differ between segments but was lower for Diet C compared to Diet G and Diet L (Diet C: 3.6, Diet G: 4.5, Diet L: 4.7, SEM = 0.23, *p* = 0.020). Valerate, iso-valerate and iso-butyrate concentrations did not differ between diets, but iso-butyrate was higher in the faeces than in the caecum (Table 5). The concentration of L-lactate was higher for Diet C compared to Diets G and L and the concentration of D-lactate was higher in the faeces than in the caecum (Table 6). Hindgut pH increased from caecum to faeces when the horses were fed Diet L in contrast to Diets C and G where pH decreased from caecum to faeces and faecal pH was higher in Diet L than in Diet G and C (Table 6). The BCs of the forages were 52 g, 35 g and 81 g lactic acid/kg DM for the early harvested grass haylage, the late harvested grass haylage and the lucerne haylage, respectively. The BC of the caecal and colon digesta was higher than for the faeces and there was a tendency for an effect of diet with numerically higher values when the horses were fed Diet G (Table 6).

## 4. Discussion

This study aimed to examine the effect of feeding young grass haylage or lucerne haylage compared to the more conventional mature grass haylage and concentrate diet on the microbial ecosystem, its activity and the BC of the equine hindgut. The three experimental diets implied differences in fibre intake; Diet G provided a lower, and Diet L a higher, intake of crude fibre, NDF, ADF, ADL and cellulose, whereas Diet C supplied the highest intake of hemicellulose.

Although differences in cellulose intake, the caecal, colonic and faecal values of cultured cellulolytic bacteria did not differ between diets. The lack of dietary effect on cellulolytic enumeration by culture was supported by the lack of effect on cellulolytic strains quantification using the real-time qPCR analysis. The absolute values of total bacteria and the cellulolytic bacteria, *F. succinogenes*, *R. flavefaciens* and *R. albus*, indeed showed no difference between the diets. The results obtained by different methods were consistent thereby confirming the observed microbial alterations due to dietary changes.

Using the real-time qPCR analysis, absolute values for total bacteria, *F. succinogenes* and *R. flavefaciens,* were higher in the faeces than in the caecum and ventral colon content. This is in contrast to previous results where similar levels of *R. flavefaciens* and *F. succinogenes* were found in the ventral colon, dorsal colon and rectum [41]. Using culturing techniques, a lack of differences in concentrations of cellulolytic bacteria between the caecum, RV colon and faeces, and higher concentrations in the caecum than the terminal colon, has been reported [13,42]. Viable total bacteria have been shown to not differ between RV colon and faeces, to have higher concentrations in the caecum than in the terminal colon, and to have lower concentrations in the caecum than in the RV colon and faeces [13]. These varying results might be due to different techniques, different diets and/or different sampling times after feeding.

Oppositely to cellulolytic, cultured xylanolytic bacteria counts were higher when horses were fed Diet C compared to Diets G and L. This was in accordance with the highest intake of hemicellulose supplied by Diet C. Higher counts of xylanolytic bacteria in pig faeces have previously been shown, and higher in vitro digestibility of hemicellulose with the faecal samples as inocula, on a high fibre diet (11.5% hemicellulose) compared to a low fibre diet (3.8% hemicellulose), indicating increases in both number and activity [43]. We measured no difference in SCFA concentrations between the diets. However, we observed a lower (acetate + butyrate)/propionate ratio in the caecum, colon and faeces when horses were fed Diet C compared to Diets G and L, suggesting a lower fibrolytic and higher amylolytic activity of the microbial communities.

Equine caecal fungi digest cellulose, hemicellulose, starch, xylan and pectin [10]. The production and secretion of xylanases are two of the major characteristics of all anaerobic fungi [44]. In contrast to ruminal strains, equine caecal strains of *Piromyces citronii* have been shown to not produce lactate [9]. In the present study, ANOVA was performed with one diet as a point of comparison and to compare the three segments. Choosing the reference is difficult and the biological interpretations of the results are complex [45]. The results showed that when Diets C and G were compared, the concentrations of fungi in faeces were higher than those in the caecum and RV colon, which was not the case with the other diet comparisons. This might indicate that Diet G favoured an increase of fungi in the faeces. Further investigations are needed to understand why the effect was more pronounced in faeces.

Total anaerobic fungal concentrations have been shown to differ between segments with the highest concentration in the RV colon followed by right dorsal colon and the lowest concentrations in the left ventral colon and rectum [46]. Another study presented both total and cellulolytic fungal concentrations that were ten-fold higher in the colon than in the caecum [11]. This is in contrast to our study, where concentrations of fungi did not differ between the caecum and the colon, but different techniques were used and data were treated differently, which complicates the comparison between the studies.

Bovine rumen and human and pig intestine pectinolytic isolates have been shown to ferment a relatively wide range of carbohydrates [47,48,49,50], but nutritionally limited pectinolytic bacteria utilising only pectin and related compounds have also been isolated [48,50,51]. Although Diet L provided the highest intake of NSP, this did not result in higher pectinolytic bacteria counts. Cell wall lucerne pectic polysaccharides have been shown to be rapidly degraded from both stems and leaves by ruminal microbes [52]. The rapid degradation of lucerne pectic polysaccharides by ruminal microbes results in a SCFA production of mainly acetate with no pH decline in the rumen buffer system [52]. Diet L also showed an inverse pattern with increasing pH from caecum to faeces compared to in Diets C and G.

The relative quantitative method was also used to quantify protozoa and there was no effect of diet or segment on hindgut protozoa and the data also showed a high individual variation. No diet effect on protozoa numbers has previously been shown comparing timothy and clover hay with or without oats, but they found a diet influence on the relative proportion of protozoa types in the caecum: *Cycloposthium bipalmatum* increased on the timothy hay diet and *Blephacorys uncinata* increased on the oat diets [17]. Higher total protozoa concentrations in the colon than in the caecum have previously been demonstrated, as well as considerable variations within individual ponies/horses [11,53].

Diet C provided a relatively small starch intake of <1 g/kg BW and day but still contained more readily digestible carbohydrates and provided the highest intake of starch between the three diets. Concentrations of L-lactate in the caecum and colon were higher when the horses were fed Diet C compared to Diets G and L. In accordance, concentrations of lactate-utilising bacteria were higher when the horses were fed Diet C, but amylolytic bacteria did not differ. However, the increase in L-lactate might indicate an increase in L-lactate producing bacteria or their activity. Three species that appear to be the predominant lactic acid producing bacteria in the equine hindgut are *Streptococcus bovis*, *Streptococcus equinus* and *Lactobacillus salivarius,* where the first two produce only L-lactate and the third highest levels of L-lactate and less D-lactate [54,55]. A shift in the (acetate + butyrate)/propionate ratio can be expected when comparing starchy diets with high fibre diets [56], and in this study it was observed as well with lower starch intake as Diet C resulted in a lower (acetate + butyrate)/propionate ratio in all segments compared to Diets G and L. Previously, no effect of diet was found on the pH of the pony caecum when comparing timothy and clover hay with or without oats, but an increase in total SCFA concentration was found when clover hay was fed [17]. In our study, the increased L-lactate concentration and lower (acetate + butyrate)/propionate ratio in the caecum and colon when the horses were fed Diet C did not have a dramatic effect on pH, which did not fall below 6.9. For D-lactate, there was an effect of segment with higher concentrations in the faeces than in the caecum. However, there was an interaction between diet and segment for pH that indicated a decrease in pH from caecum to faeces on Diets C and G and an opposite increase in pH from caecum to faeces on Diet L. Pectin fermentation renders no lactic acid output [57] and has the galacturonic acid structure providing potential buffering through cation exchange and metal ion binding [5]. The increase in pH from caecum to faeces when the horses were fed Diet L might be due to the lack of lactate production and the higher buffering capacity of the lucerne forage. However, although the lucerne haylage had the highest BC of the feeds, when the diets had reached the caecum there was a tendency for a higher BC of Diet G. The BC of the caecum and colon digesta was five to fifteen-fold higher than the BC of the feeds, suggesting that the effect of the initial BC of the feeds decreased as the digesta travelled through the digestive tract. In sheep, the BC of caecal digesta was shown to be nearly double than the BC of rumen digesta [58]. The high post-ileal BC may be explained by the endogenous addition of HCO_3_ to small intestinal content [59]. The higher BC of the lucerne haylage still might have had an effect in the stomach (not measured) as diets including alfalfa have previously been suggested to buffer stomach acids in horses [19] and pigs [60]. Important to note is that dietary regimens can probably affect the BC along the entire gastrointestinal tract. as previously shown with a higher BC in faeces when hay was fed prior to concentrate than vice versa [20]. The BC was 4.5 to nine-fold higher in the caecum and colon than faeces and may be explained by the great absorption of electrolytes, organic acids and water in the distal parts of the large intestine prior to faecal excretion [59,61].

The substantial differences in the stage of maturity between the grasses, the IVDOM values of 89% and 63% for the young and mature grass, respectively, resulted in differences in fibre intake and probably in fibre digestibility, but did not have a major impact on microbial numbers. However, Diet G resulted in lower L-lactate than Diet C and, numerically, the highest BC. Early harvested forage providing high energy without the negative effects of concentrate opens up the possibility for forage-only diets for high performing horses.

## 5. Conclusions

Differences in DM and fibre intakes due to early and late harvested grass and legume did not change the concentrations of cellulolytic and total bacteria in the hindgut flora. In contrast, even a small starch intake of <1 g/kg BW and day seemed to have an effect on lactate-utilising bacteria, L-lactate concentrations and the (acetate + butyrate)/propionate ratio in the caecum and colon. Although the initial BC of different forages probably has an impact on the stomach environment, there seemed to be less effect on the hindgut digesta. The effects and importance of fibrous diets on equine hindgut fungi and protozoa needs further investigation. However, we conclude that a forage diet that fulfils the energy and protein requirements, without having to add starch rich concentrate, can benefit hindgut health.

## Figures and Tables

**Table 1:** Intake of DM, energy and dietary components of the three diets ^1^ in g/100 kg BW and day if not otherwise stated (Least square means with the pooled standard error of the mean).

	Diet C	Diet G	Diet L	SEM	*p*-Values
DM (kg/100 kg BW and day)	1.4 ^a^	1.2 ^b^	1.4 ^a^	0.04	0.001
Energy (MJ/100 kg BW and day) ^2^	12.1 ^ab^	12.5 ^a^	11.7 ^b^	0.39	0.032
Crude protein	181 ^a^	199 ^b^	191 ^a,b^	5.8	0.016
Crude fibre	420 ^a^	337 ^b^	499 ^c^	15.4	<0.001
Neutral detergent fibre	775 ^a^	617 ^b^	742 ^a^	23.3	0.001
Acid detergent fibre	465 ^a^	362 ^b^	544 ^c^	15.8	<0.001
Acid detergent lignin	54 ^a^	38 ^b^	97 ^c^	3.1	<0.001
Hemicellulose ^3^	309 ^a^	256 ^b^	197 ^c^	7.7	<0.001
Cellulose ^3^	407 ^a^	329 ^b^	446 ^c^	12.9	<0.001
Non-structural carbohydrates ^4^	316 ^a^	228 ^b^	301 ^a^	9.2	<0.001
Non-starch polysaccharides ^4^	161 ^a^	174 ^a^	204 ^b^	9.8	0.016
Starch	91 ^a^	4 ^b^	16 ^c^	1.8	<0.001
Water soluble carbohydrates ^5^	61 ^a^	53 ^b^	21 ^c^	2.1	<0.001
Glucose	11 ^a^	10 ^a^	6 ^b^	0.4	<0.001
Fructose	21 ^a^	24 ^b^	9 ^c^	0.6	<0.001
Sucrose	13 ^a^	5 ^b^	0.1 ^c^	0.4	<0.001
Fructans	9 ^a^	6 ^b^	5 ^c^	0.6	0.010
Maltodextrins	7	6	3	1.9	0.131
Calcium	17 ^a^	18 ^b^	21 ^c^	0.6	<0.001
Phosphorus	3	3	3	0.1	0.235
Magnesium	3 ^a^	3 ^a^	5 ^b^	0.2	<0.001
Sodium	4 ^a^	4 ^b^	4 ^a^	0.1	0.010
Potassium	25 ^a^	33 ^b^	34 ^b^	1.3	0.001

^1^ Diet C: concentrate (oats, soybean meal) and late harvested grass haylage (35:65 energy ratio), Diet G: early and late harvested grass haylage (80:20) (+small amount soybean meal), Diet L: lucerne haylage and late harvested grass haylage (80:20). ^2^ The estimated energy values, in mega joule, of the forages were calculated from the in vitro digestible organic matter values [22]. ^3^ Hemicellulose and cellulose concentrations in the feeds were calculated by weight difference: NDF-ADF and ADF-ADL, respectively. ^4^ Non-structural carbohydrate and non-starch polysaccharide concentrations in the feeds were calculated NSC = 100 − (NDF + protein + fat + ash) and NSP = NSC − (starch + sugars), respectively [5]. ^5^ Free glucose, free fructose, sucrose, fructans and maltodextrins. ^a,b,c^ Mean values within a row with unlike superscript letters were significantly different (*p* < 0.05).

**Table 2:** Sequences of primers used for real-time PCR.

Target	Forward Primer	Reverse Primer	Reference
Total bacteria	5′-CGGCAACGAGCGCAACCC-3′	5′-CCATTGTAGCACGTGTGTAGC-3′	[34]
*Fibrobacter succinogenes*	5′ GTTCGGAATTACTGGGCGTAAA 3′	5′ CGCCTGCCCCTGAACTATC 3′	[35]
*Ruminococcus flavefaciens*	5′ CGAACGGAGATAATTTGAGTTTACTTAGG 3′	5′ CGGTCTCTGTATGTTATGAGGTATTACC 3′	[34]
*Ruminococcus albus*	5′ CCCTAAAAGCAGTCTTAGTTCG 3′	5′ CCTCCTTGCGGTTAGAACA 3′	[36]
Fungi	5′-GAGGAAGTAAAAGTCGTAACAAGGTTTC-3′	5′-CAAATTCACAAAGGGTAGGATGATT-3′	[34]
Protozoa	5′-GCTTTCGWTGGTAGTGTATT-3′	5′-CTTGCCCTCYAATCGTWCT-3′	[35]

**Table 3:** Microbial counts (log colony forming units/mL or g) of equine caecum and colon (right ventral) contents and faeces after three weeks of adaptation to forage based diets differing in fibre composition and maturity ^1,2^.

	Diets			SEM	*p*-Values Diet
	Diet C	Diet G	Diet L		
Total anaerobic bacteria	8.1	7.5	7.7	0.21	0.182
Pectinolytic bacteria	7.7 ^a^	7.0 ^b^	7.2 ^b^	0.15	0.022
Xylanolytic bacteria	7.6 ^a^	6.7 ^b^	7.1 ^b^	0.15	0.011
Cellulolytic bacteria	5.7	5.5	5.7	0.22	0.846
Amylolytic bacteria	6.0	5.7	5.3	0.27	0.202
Lactate-utilising bacteria	7.4 ^a^	6.4 ^b^	6.6 ^b^	0.18	0.007

^1^ Since there were no diet × segment interactions, least square means across segments (with the pooled standard error of the mean) are presented (*n* 5). ^2^ Diet C: concentrate (oats, soybean meal) and late harvested grass haylage (35:65 energy ratio), Diet G: early and late harvested grass haylage (80:20) (+small amount soybean meal), Diet L: lucerne haylage and late harvested grass haylage (80:20). ^a,b^ Mean values within a row with unlike superscript letters were significantly different (*p* < 0.05).

**Table 4:** The relative quantification of fungi and protozoa in caecum, colon (right ventral) and faeces using the comparative critical threshold (∆∆Ct) method with data presented as the N-fold microbe expression difference between diets (Diet C vs. G with Diet L as a reference, Diet C vs. L with Diet G as a reference and Diet G vs. L with Diet C as a reference) ^1,2^.

	Segment			SEM	*p*-Value Segment
	Caecum	Colon	Faeces		
Fungi					
Diet C vs. G	1.33 ^a^	2.06 ^a^	14.25 ^b^	3.337	0.014
Diet C vs. L	1.12	0.70	1.97	0.639	0.369
Diet G vs. L	9.17	1.40	0.16	3.900	0.208
Protozoa					
Diet C vs. G	1.15	1.17	2.41	1.140	0.660
Diet C vs. L	1.02	3.17	2.37	0.943	0.346
Diet G vs. L	1.89	1.00	2.24	0.775	0.566

^1^ Since there were no effect of diet and no diet × segment interaction, least square means across diets (with the pooled standard error of the mean) are presented (*n* 5). ^2^ Diet C: concentrate (oats, soybean meal) and late harvested grass haylage (35:65 energy ratio), Diet G: early and late harvested grass haylage (80:20) (+small amount soybean meal), Diet L: lucerne haylage and late harvested grass haylage (80:20). ^a,b^ Mean values within a row with unlike superscript letters were significantly different (*p* < 0.05).

**Table 5:** Equine caecal, colon (right ventral) and faecal SCFA concentrations (mmol/l) after three weeks of adaptation to forage based diets differing in fibre composition and maturity ^1,2^.

	Segment			SEM	*p*-Value Segment
	Caecum	Colon	Faeces		
Total SCFA	60.4 ^a^	64.7 ^a^	38.3 ^b^	3.91	<0.001
Acetate	41.9 ^a^	45.2 ^a^	26.7 ^b^	2.44	<0.001
Propionate	11.9 ^a^	12.4 ^a^	6.9 ^b^	0.95	0.001
Butyrate	5.0 ^a^	5.1 ^a^	2.8 ^b^	0.43	0.003
Valerate	0.8	0.6	0.4	0.15	0.134
Iso-butyrate	0.3 ^a^	0.6 ^a,b^	0.8 ^b^	0.11	0.038
Iso-valerate	0.6	0.8	0.7	0.13	0.245

^1^ Since there were no effects of diet and no diet × segment interactions, least square means across diets (with the pooled standard error of the mean) are presented (*n* 5). ^2^ Diet C: concentrate (oats, soybean meal) and late harvested grass haylage (35:65 energy ratio), Diet G: early and late harvested grass haylage (80:20) (+small amount soybean meal), Diet L: lucerne haylage and late harvested grass haylage (80:20). ^a,b^ Mean values within a row with unlike superscript letters were significantly different (*p* < 0.05).

**Table 6:** Equine caecal, colon (right ventral) and faecal D and L-lactate, pH and buffering capacity (BC) after three weeks of adaptation to forage based diets differing in fibre composition and maturity ^1,2^.

	Diets			SEM	*p*-Values		
	Diet C	Diet G	Diet L		Diet	Segment	Diet × Segment
**D-lactate (mmol/L)**							
Caecum	0.8	0.6	0.3	0.37	0.174	0.025	0.111
Colon	1.6	0.4	0.8				
Faeces	1.4	1.9	1.0				
**L-lactate (mmol/L)**							
Caecum	1.1	0.5	0.3	0.23	0.022	0.640	0.127
Colon	1.3	0.3	0.7				
Faeces	0.7	0.7	0.6				
**pH**							
Caecum	7.0 ^A^	7.0 ^A^	7.0 ^A^	0.08	0.012	0.027	0.002
Colon	6.9 ^A,B^	7.0 ^A^	7.1 ^A,B^				
Faeces	6.7 ^a,B^	6.5 ^a,B^	7.2 ^b,B^				
**BC (g lactic acid/kg dry digesta)**							
Caecum	353	590	387	92.4	0.071	<0.001	0.499
Colon	513	709	471				
Faeces	78	77	48				

^1^ Values are least square means with the pooled standard error of the mean, *n* 5. ^2^ Diet C: concentrate (oats, soybean meal) and late harvested grass haylage (35:65 energy ratio), Diet G: early and late harvested grass haylage (80:20) (+small amount soybean meal), Diet L: lucerne haylage and late harvested grass haylage (80:20). ^a,b^ Mean values within a row with unlike superscript letters were significantly different (*p* < 0.05). ^A,B^ Mean values within a parameter and column with unlike superscript letters were significantly different (*p* < 0.05).

## Data Availability

Data are available under request to the authors.
